# Effects of permeability, grid resolution, and carbonate reactions on CO_2_ distribution and sulphate scaling in seawater-flooded carbonates

**DOI:** 10.1038/s41598-025-14504-z

**Published:** 2025-08-11

**Authors:** Ali M. Al-Behadili, Eric J. Mackay

**Affiliations:** Heriot-Watt University, Edinburgh, UK

**Keywords:** Brine mixing, Reservoir behaviour, Carbonate scales, Sulphate scale, Chemical reaction, Temperature dependence, CO_2_ solubility, Inorganic chemistry, Engineering

## Abstract

During oilfield production, mineral scale deposition in production wells and surface facilities presents major difficulties, particularly when water breakthrough occurs. Hard sulphate scales, like barite, are frequently the result of incompatible interactions between the formation and injection waters. In contrast, carbonate scales are caused by variations in the temperature, pressure, pH, composition of the brine, and the amounts of CO_2_ in the aqueous and hydrocarbon phases. In waterflooded reservoirs where injection water composition can be controlled, this study investigates the effects of temperature, ionic concentration, pH, and CO_2_ availability on the risks of carbonate and sulphate scaling. Particularly, scaling hazards in carbonate-rich formations are greatly influenced by precipitation of magnesium-rich carbonate. The significance that CO_2_ partitioning from hydrocarbons into injected saltwater plays in scaling estimates has been ignored in earlier studies. To close this gap, this study investigates how temperature and reservoir pressure affect oil recovery and scale management, particularly in systems that dip below bubble point pressure. A commercial reservoir simulator, which couples aqueous and mineral geochemistry with three-phase fluid flow calculations, has been used in this study. Equilibrium reactions have been considered in three-dimensional (3D) models. Oilfield data have been used to identify the parameters of significance to consider in the calculations, such as ionic concentrations, hydrocarbon composition, mineral components etc. Key findings, importantly, the results identify that for the reservoir temperature of 100 °C considered and for the primary mineral assemblage, calcite dissolution and magnesium-rich carbonate precipitation are interdependent. They are affected by the abundance of CO_2_ in the residual oil phase, and this evolves over time, impacting the concentration of calcium and magnesium in the brines traversing the reservoir. Temperature changes around the injection wellbore also impact component and mineral solubilities, especially in terms of anhydrite and gypsum reactions. All these factors impact the calcium, magnesium, barium, strontium, sulphate and bicarbonate concentrations at the production well, and hence the scaling risk in the production system. In conclusion, managing reservoir conditions such as temperature, ionic concentrations, and CO_2_ distribution is important for reducing the likelihood of scale deposition while preserving oil recovery in carbonate-rich, water-flooded reservoirs.

## Introduction

**Fig. 1:**
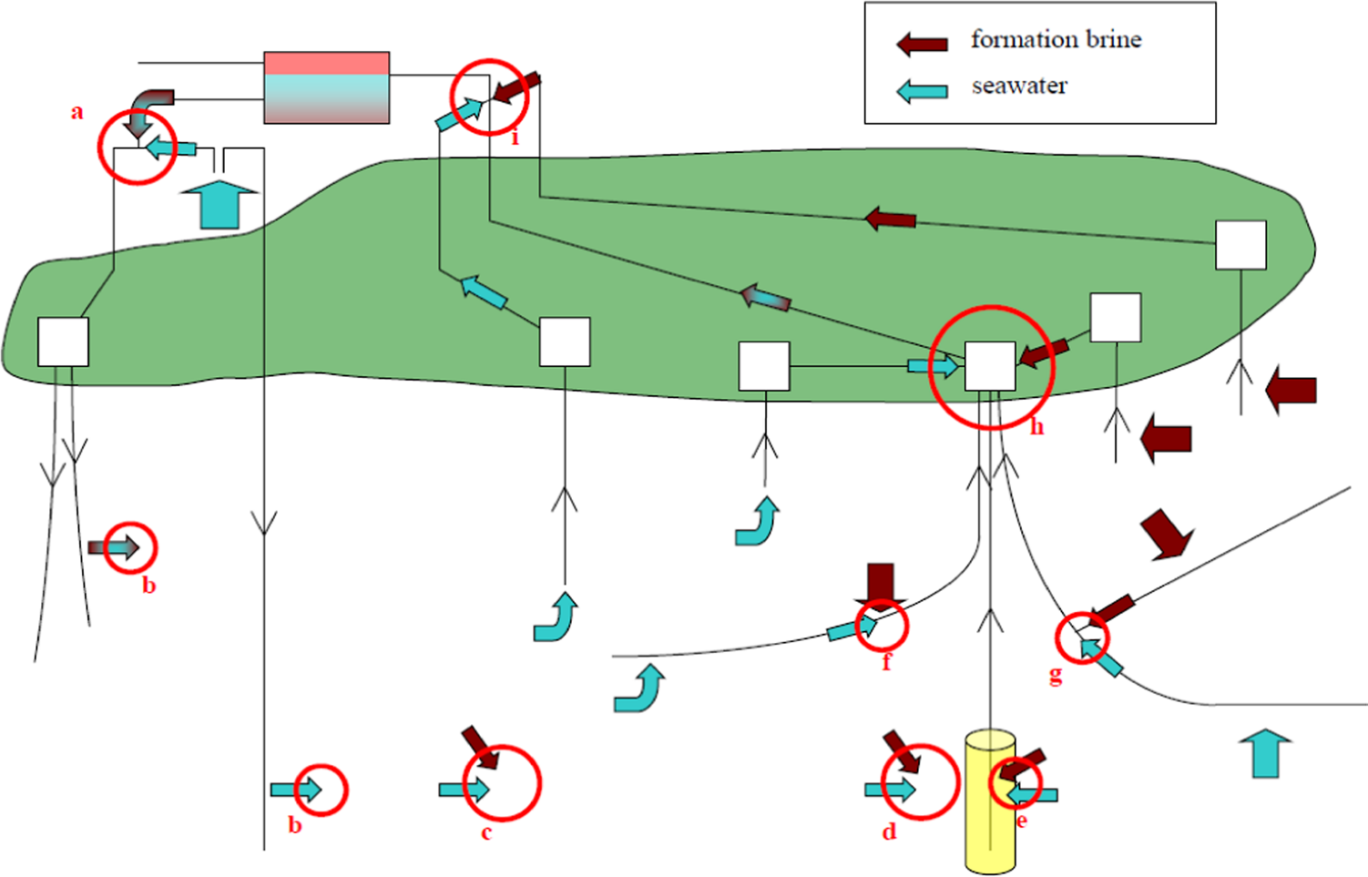
Location throughout the flow system where brine mixing may take place: (a) where produced water and seawater are mixed at surface prior to injection; (b) where injection brine enters the formation contacting formation water; (c) deep within the reservoir; as flow streams carrying different brines converge (d) on approach to the production wells but outside the scale inhibitor squeezed zone (denoted in yellow), (e) inside the squeezed zone, (f) inside the completion, (g) at the junction of a multilateral well, (h) at a seabed manifold or surface collection point, and (i) at the platform or other surface facilities before separation. After Mackay^8^.

One method to increase oil recovery from subsurface reservoirs is water flooding; this entails injecting water to sweep oil toward the production wells and maintain reservoir pressure, such that there is sufficient energy to lift fluids through the production well to the surface. However, there may be various challenges associated with applying this method. Managing scale formation is one such challenge and is regularly considered a severe issue for the oil and gas industry. Many studies^1–4^ show that scale formation can be caused by either the mixing of waters (e.g., barium sulphate, BaSO_4_) or by changes in temperature and pressure due to production (e.g., calcium carbonate, CaCO_3_). Scale problems may be initiated due to the difference in ionic compositions between formation (connate and aquifer) waters and injection water (mostly seawater): many chemical reactions will take place because of mixing between these brines^5,6^. Serious issues, such as decreased oil output and significant increases in operational costs, may arise if a scale management regime is not correctly implemented, potentially leading to the blocking of perforations, casing, valves, tubing, and other critical components^7^. The sulphate scales are more difficult to prevent or remove because their solubilities in the aqueous phase are very low due to high thermodynamic stability. On the other hand, carbonate scales are more soluble and can be removed by injection of a suitable acid or may be prevented by the application of scale inhibitors. Carbonate scales usually deposit downstream in wellbores, pipelines, and surface facilities, while sulphate scales may also occur in various places inside the reservoir, wellbore, and downstream, as depicted in Fig. 1^7,8^. By contrast with many other scales, for calcium sulphate (which at temperatures above 90 °C usually is in the form of anhydrite), the solubility decreases very rapidly as the temperature increases^9^.

Sulphate scale precipitation and the evolving scale risk in the wells depend on many factors associated with brine flow and mixing, and with chemical interactions: mobility ratio, oil/water interfacial tension (IFT), temperature, the degree of mixing and precipitation in the reservoir, and the inhibitor adsorption levels to name a few^10,11^. When waters mix at the start of water injection (with the injection brine often containing high sulphate concentrations and the formation brine being rich in barium, calcium, and/or strontium ions) BaSO_4_, CaSO_4,_ and/or SrSO_4_ precipitation may take place. Of these scales, the solubility of BaSO_4_ is the lowest^12^, and so it is typically the most problematic to address.

For severely scaling sulphate systems where seawater is the injected brine, it is very important to assess the potential benefit and cost of Low Sulphate Seawater (LSSW) injection in contrast to full sulphate seawater (FSSW) injection to reduce the deposition risks of all the sulphate scales. (LSSW injection may also reduce souring risk—a topic *not* addressed in this paper, but nonetheless of importance in some settings.) Injection of LSSW is achieved by using a Sulphate Reduction Plant (SRP) to reduce the concentration of sulphate in the injection brine. While in some fields the practice has been to use the SRP throughout the entire injection period, increasingly it is becoming important to consider whether or not it is possible to switch from LSSW to FSSW, or more usually vice-versa, during the lifetime of the field, and if so, when would be the best time to make the change to decrease the operational cost (Opex)^13,14^.

Another advantage of reducing the requirement for the SRP is a reduction in the carbon footprint of the operation, since the plant requires power to function. Such decisions may depend on whether onshore or offshore fields are being considered, and in the case of the latter, the water depth is important, since desulphation of seawater may be more economic for deep subsea wells where interventions (such as squeezes) are expensive and difficult to apply^15^.

In performing assessments of scaling risk and impact of brine compositions, thermodynamic calculations must be carried out. The scale precipitation tendency is typically used to identify the scaling risk, and is expressed using the Saturation Ratio or as the Saturation Index (= Log_10_(Saturation Ratio)); Table 1 shows the interpretation of the Saturation Ratio and Saturation Index^16^.

**Table 1:** Description of the state of a brine relative to a given mineral.

Saturation ratio (SR)	Saturation Index = (log_10_(SR))	Scale prediction
< 1.0	< 0.0	Brine is undersaturated and, where present, minerals will dissolve
= 1.0	= 0.0	Brine is saturated, and minerals will neither form nor dissolve
> 1.0	> 0.0	Brine is oversaturated, and scale will tend to precipitate

While studying scale problems, it is very important to provide insights into what components are involved in the in situ geochemical reactions that take place while the brines are displaced through the reservoir^17^. Furthermore, how the precipitation and dissolution of minerals and the ion exchange reactions occur within the reservoir can be identified^18^. Among the various types of mineral scales, calcium sulphate stands out as a major concern in carbonate reservoirs, capable of causing severe flow assurance and formation damage issues due to the large mass that can deposit. Several parameters contribute to this problem, including temperature, pressure, fluid concentration, the ratio of brine to hydrocarbon, fluid dynamics, and the type of porous medium^19^.

Scale deposition may have a significant impact on the petrophysical properties of the reservoir in the near production wellbore area. For instance, one author reported in core flood experiments that the permeability of a sandstone core before seawater injection was 80 mD, and that after injecting two pore volumes of seawater (60 mL) the permeability was reduced to 63 mD, which means a loss of about 20% in permeability^20,21^. When such tests are performed, it is necessary to check that no other types of formation damage are occurring. This is necessary since a simple spreadsheet calculation of material balance will identify that even if every single available scaling cation in the original formation brine in the core were to react with a corresponding injected sulphate ion, the total change in porosity would typically be less than 0.01%^5^. (Clearly, such complete mixing is something that is impossible as formation water will be displaced out of the core before being contacted by injection water.) As Fig. 1 illustrates, the scaling problems occur when there is a convergence of brine streams and there are many (usually hundreds or thousands) of pore volumes mixing in a given location in the reservoir: such a location would typically be near production wells, not injection wells nor in the middle of the reservoir.

## Materials and methodology

### 3D model construction

Numerical modelling studies have been conducted on a simple 3D quarter five-spot model of a waterflood run using the CMG GEM compositional and geochemical reservoir simulation software from Computer Modelling Group, Calgary, Canada) (CMG, 2025). The synthetic model is developed using literature and field data to study scale problems in both carbonate and sulphate scaling systems and to identify the effect of many parameters and variables that have a relationship with scale precipitation. The model geometry is kept deliberately simple (quarter five-spot) so that this study can focus on the impact of various sensitivities to geochemical parameters, without having to address the question of whether or not observed behaviours are the consequence of complex flow dynamics due to heterogeneities, reservoir features such as faults, baffles, and pinchouts, or due to interferences between multiple injection and production wells. In future work, the understanding and insights gained from this paper will be applied in full-field simulation studies, where the richness of these complexities will also be addressed.

The description of the 3D model system is summarised in Table 2 below.

**Table 2:** Model reservoir properties and well controls.

Property	Values
Cartesian grid dimensionality	9 × 9 × 4 cells
Grid cell sizes (uniform)	175 m × 175 m × 10 m
Horizontal permeability (homogenous)	200 mD
Vertical permeability (homogenous)	20 mD
Porosity (homogenous)	0.2
Reservoir depth (top)	3000 m
Pressure @ 3000 m	29,500 kPa
Temperature @ 3000 m	100 °C
Initial saturations	Swi = 0.21; Soi = 0.79; Sgi = 0
Production well liquid rate control	600 m^3^/day
Injection well water rate control	1000 m^3^/day
Injection well maximum pressure limit	40,000 kPa
Injection water temperature	40 °C

The aqueous phase density is calculated using the Rowe-Chou correlation^22^, and the aqueous phase viscosity using the Kestin, Khalifa and Correia correlation^23^. The simulation is run for 40 years.

A seven-component Peng-Robinson Equation of State (EoS) fluid model is used. The initial liquid hydrocarbon phase composition is shown in Table 3. It was selected based on typical hydrocarbon compositions in the Brazilian pre-salt reservoirs in the Santos Basin^24^, which contain CO_2_, and thus where the partitioning of CO_2_ from oil to injected brine will impact pH and geochemical reactivity.

**Table 3:** Initial oil composition.

Components	Initial global mole fraction
‘CO_2_’	0.0611
‘N_2_toC_1_’	0.5252
‘C_2_toNC_4_’	0.1523
‘IC_5_toC_10_’	0.1146
‘C_11_toC_14_’	0.0586
‘C_15_toC_16_’	0.0881
‘H_2_O’	0.0001

A vertical water injection well is located in cells (1,1,1-4) and a vertical production well is located in cells (9,9,1-4), at the opposite corner of the model from the injection well.

The aqueous phase is modelled as containing 11 components (Ba^2+^, SO_4_^2−^, Ca^2+^, Sr^2+^, Mg^2+^, Na^+^, Cl^−^, H^+^, CO_3_^2−^, OH^−^ and HCO_3_^−^). These components are chosen because they are required to model the most common carbonate (CaCO_3_ and Ca.nMg.(n + 1)CO_3_) and sulphate (BaSO_4_, CaSO_4,_ and SrSO_4_) scales that occur when producing reservoir brines^26^. Their concentrations in the formation and injection brine are presented later in the section on geochemical results.

Table 4 shows the scenarios or cases that have been included in this study; this paper addresses the effect of permeability distribution (heterogeneity effect—all cases coarse scale) in four cases and the impact of grid resolution in another four scenarios (grid resolution effect—all cases homogeneous).

**Table 4:** Scenarios included in this study, addressing impact of heterogeneity and grid resolution effects.

Heterogeneity effect		Grid resolution effect	
Scenario	Horizontal permeability (md)	Scenario	Number of cells
Homogenous	200 (base case)	Low resolution	9 × 9 × 4 (base case)
Coarsening-up	350, 250, 150, 50	Medium low resolution	27 × 27 × 12
Coarsening-down	50, 150, 250, 350	Medium high resolution	45 × 45 × 20
Layered	350, 50, 150, 250	High resolution	90 × 90 × 40

**Fig. 2:**
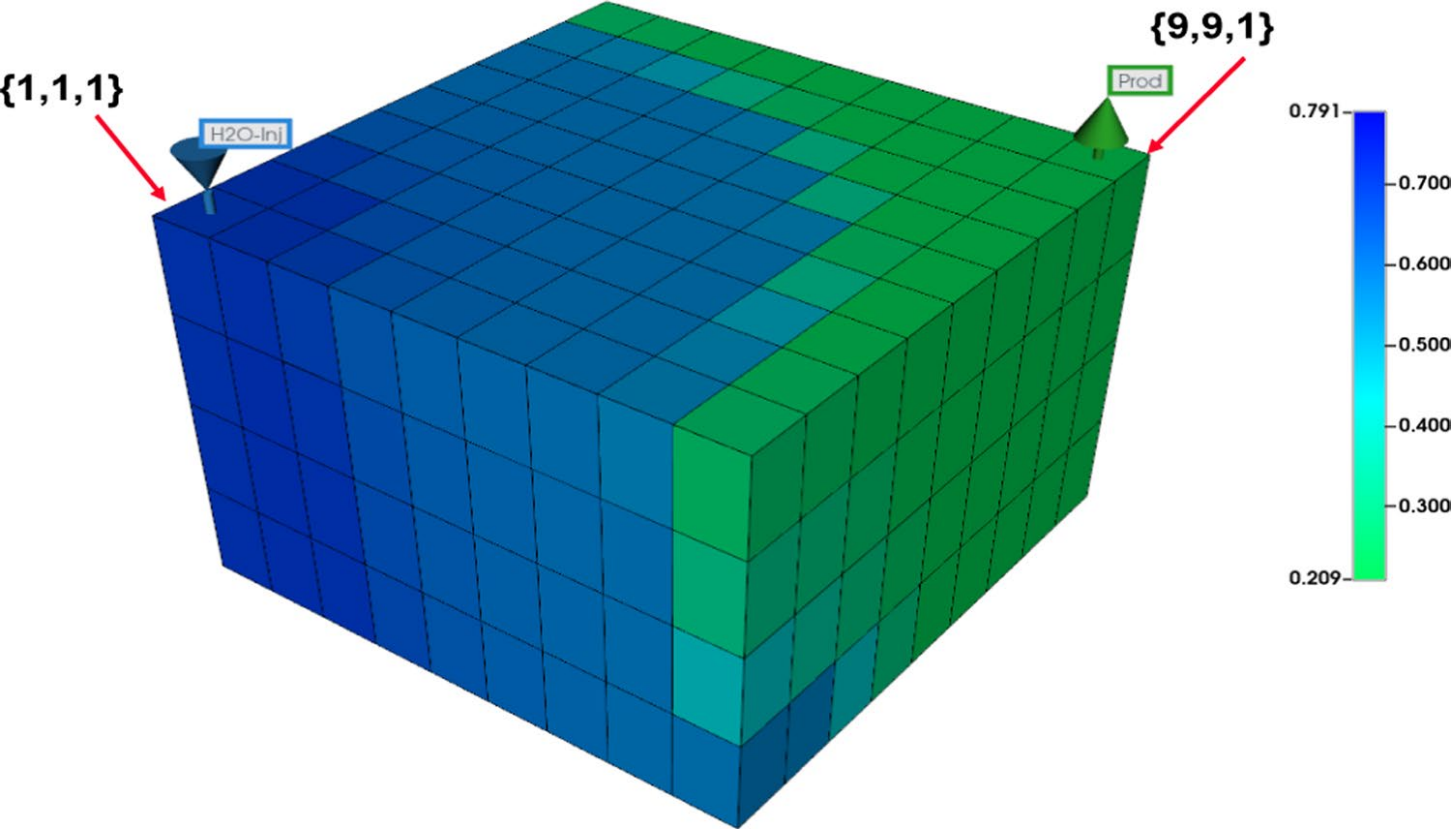
3D view of water saturations in the homogenous low-resolution model shortly before water breakthrough.

Figure 2 illustrates the grid geometry and well locations, as well as the water phase distribution shortly before water breakthrough for the homogeneous low-resolution model. Due to the relatively high flow rates, gravity has only a small effect, with a slightly higher oil saturation towards the top of the system.

**Fig. 3:**
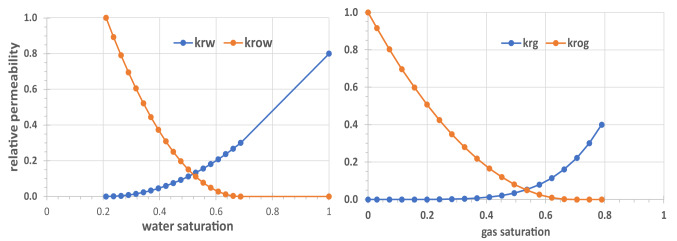
Water–oil (left) and gas-oil (right) relative permeability functions.

Relative permeability functions are shown in Fig. 3. The system is an offshore carbonate reservoir that is being considered for CO_2_ EOR and is mildly water-wet. Three-phase relative permeabilities are calculated using Stone’s second method, with modifications by^26^. Capillary pressure is not modelled.

### Objectives and methodology

The overall research plan involves the use of commercial reactive transport software as a tool to model fluid flow and mineral reactions in a range of scenarios and test various hypotheses about the impact of various injection water treatment options. As mentioned above, the objective of this part of the study was to investigate the effect of permeability distribution (heterogeneity effect) and grid cell sizes (grid resolution effect), as described in Table 4. The first part of this study addressed the effect of CO_2_ partitioning from the oleic phase, aqueous reactions and pH on the reservoir fluid properties and their impact on the injector and producer wells. Later work took into consideration the effect of sulphate and carbonate mineral reactions, in particular barite and calcite precipitation and dissolution. The remaining work will address what changes will occur due to the impact of temperature on anhydrite and gypsum mineral reactions.

### Mineralogy and geochemical reactions

To simulate the geochemical reactivity, both aqueous and mineral reactions are modelled.

### Aqueous reactions

Three aqueous reactions are included to model the carbonate system and capture the pH changes that will occur. These reactions are always treated as equilibrium reactions by the model.

1 $${\text{OH}}^{ - } + {\text{H}}^{ + } \leftrightarrow {\text{H}}_{2} {\text{O}}$$

2 $${\text{CO}}_{3}^{2 - } + {\text{H}}^{ + } \leftrightarrow {\text{HCO}}_{3}^{ - }$$

3 $${\text{CO}}_{2} + {\text{H}}_{2} {\text{O}} \leftrightarrow {\text{H}}^{ + } + {\text{HCO}}_{3}^{ - }$$

### Mineral reactions

Mineral reactions are based on the primary minerals initially present (only calcite is reactive) and the secondary mineral reactions specified. The secondary reactions are chosen based on the possible minerals involving the ions modelled (and measured in the brine analysis) and field experience. Their solubilities are derived from established databases (WATEQ4F^27^, MINTEQ^28^ and LLNL^29^). Since the simulations performed are non-isothermal, temperature dependence of the various mineral equilibrium constants are calculated: for instance, anhydrite, calcite and dolomite solubilities all decrease with increasing temperature within the temperature ranges encountered in this system (as identified in above databases and^30^).

- Initial minerals
  4 $${\text{Calcite:}}\quad {\text{Ca}}^{2 + } + {\text{HCO}}_{3}^{ - } \leftrightarrow {\text{H}}^{ + } + {\text{CaCO}}_{3}$$
  occupying 10% of bulk volume (exact value not significant as calcite is never completely consumed in any grid blocks)
- Secondary mineral reactions are also included.
  5 $${\text{Dolomite:}}\quad {\text{Ca}}^{2 + } + {\text{Mg}}^{2 + } + 2{\text{HCO}}_{3}^{ - } \leftrightarrow 2{\text{H}}^{ + } + {\text{CaMg}}({\text{CO}}_{3} )2$$
  (To represent dolomite or similar calcium magnesium carbonate mineral)
  6 $${\text{Anhydrite:}}\quad {\text{Ca}}^{2 + } + {\text{SO}}_{4}^{2 - } \leftrightarrow {\text{CaSO}}_{4}$$
  (precipitation at reservoir temperature, not at injection temperature)
  7 $${\text{Gypsum:}}\quad {\text{Ca}}^{2 + } + {\text{SO}}_{4}^{2 - } + 2{\text{H}}_{2} {\text{O}} \leftrightarrow {\text{CaSO}}_{4} \cdot 2{\text{H}}_{2} {\text{O}}$$
  (precipitation as the temperature approaches injection temperature)
  8 $${\text{Barite:}}\quad {\text{Ba}}^{2 + } + {\text{SO}}_{4}^{2 - } \leftrightarrow {\text{BaSO}}_{4}$$
  9 $${\text{Celestite:}}\quad {\text{Sr}}^{2 + } + {\text{SO}}_{4}^{2 - } \leftrightarrow {\text{SrSO}}_{4}$$

The mineral reactions are selected based on the Ba^2+^, Sr^2+^, Ca^2+^, and HCO_3_^−^ concentrations in the formation brine (see Table 4 later in the paper) and the SO_4_^2-^ and Mg^2+^ concentrations in the injection water and are all assumed to be equilibrium reactions, with the equilibrium constants being calculated as a function of temperature. The Pitzer activity model is used. Harvey’s correlation is used to calculate Henry’s constant, which is then used to calculate the solubility of CO_2_ in the aqueous phase. Henry’s constant is thus calculated as a function of pressure, temperature and salinity. This correlation supplied is applicable up to 150 °C and 70,000 kPa.

## Results and discussion

### Fluid flow in base case homogeneous model

The first set of calculations presented here address water injection supported hydrocarbon recovery in a 3D 3-phase quarter-five spot (one injector and one producer) model (see Fig. 2).

**Fig. 4:**
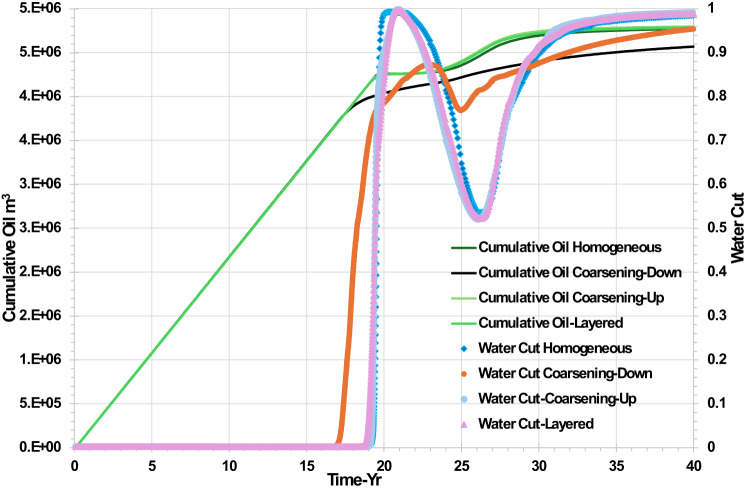
Cumulative oil and water cut in the producer well versus time for various permeability distributions.

Figure 4 shows the cumulative oil and water cut behaviour over time for the various permeability field scenarios. From the plot, we can see that while the oil production was supported by water injection from day one, water breakthroughs did not occur until after 17 years in the coarsening down scenario and around year 19 in the rest of the heterogeneity scenarios, at which time the oil production rate declined quite gradually. From Fig. 4, in the coarsening down scenario the lowest cumulative oil production was calculated due to the highest permeability in the lowest layer combining with the effect of gravity and leading to water being displaced toward the producer through the lowest layer the fastest of all the scenarios.

**Fig. 5:**
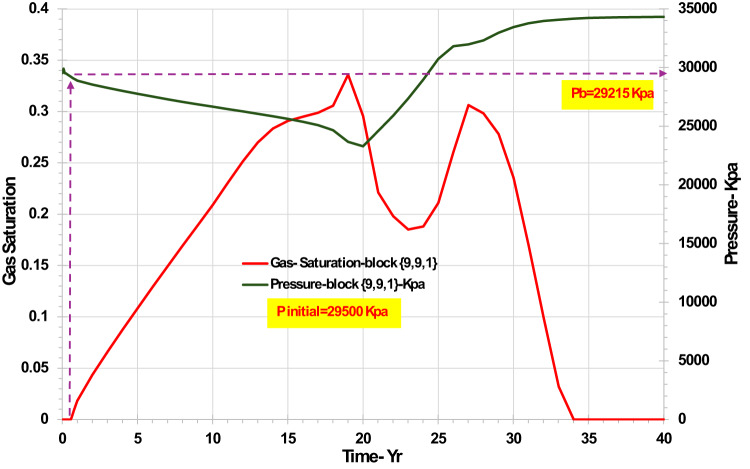
Pressure (relative to original bubble point pressure, Pb) and gas saturation in the top layer producer grid block {9,9,1} versus time—homogeneous case.

The drop in water cut some five years after water breakthrough in all cases is due to gas that had evolved from solution within the reservoir being displaced into the well by the advancing waterflood front. Thus, although the reservoir was undersaturated at the start of production, it was only just undersaturated, and due to declining pressure during production, gas evolved from solution (Fig. 5). However, after water breakthrough the reservoir pressure increased again, and as well as the water flood displacing some of the evolved gas into the production well, gas also re-dissolved back into the oil phase, so that by year 35 there was no free gas remaining in the system. The evolution of the gas during depressurisation displaced some otherwise unrecovered (mainly attic) oil, leading to an *increasing* oil recovery between year 23 and year 26; thereafter, oil production continued, but only moderate volumes were produced until the end of field life. Since the hydrocarbon system contains CO_2_, this behaviour of the gas phase is vital to understand the changing produced brine composition, pH, and the scaling tendency in the production well.

**Fig. 6:**
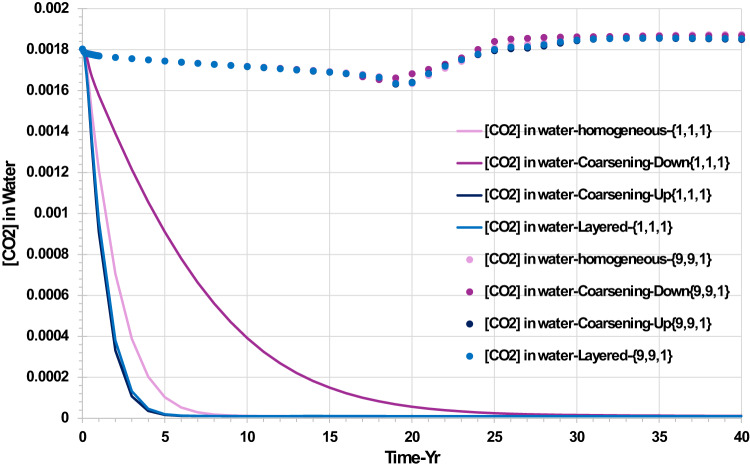
CO_2_ concentration (mole fraction) in water in the top layer injector grid block {1,1,1} and the top layer producer grid block {9,9,1} versus time—permeability distribution scenarios.

In Fig. 6 we can see that the CO_2_ content in the aqueous phase in the top layer around the production well (block {9,1,1}) varies a little (minimum mole fraction of 0.0016 to maximum of 0.0019) for all four scenarios (heterogeneity effects), depending mainly on the pressure of the grid block, but also on the presence or absence of free gas. The CO_2_ content in the oil around the injection well (block {1,1,1}) continually decreases, as CO_2_ partitions from the oil into the injected water (which has almost no CO_2_ when injected). From the figure it is observable that the CO_2_ remains available at the top of the reservoir for the longest time in the coarsening down case, for around 26 years, since the top of the reservoir has the lowest permeability. This means that gravitational and viscous forces combine to minimise the water throughput at the top of the reservoir, and therefore there is the greatest delay in the depletion of CO_2_ from the residual oil. On the other hand, the fastest CO_2_ depletion in the top layer occurs in the coarsening up and layered scenarios—both taking around 5 years. We conclude from this that permeability distribution has a significant impact on availability of CO_2_ to partition into the aqueous phase.

**Fig. 7:**
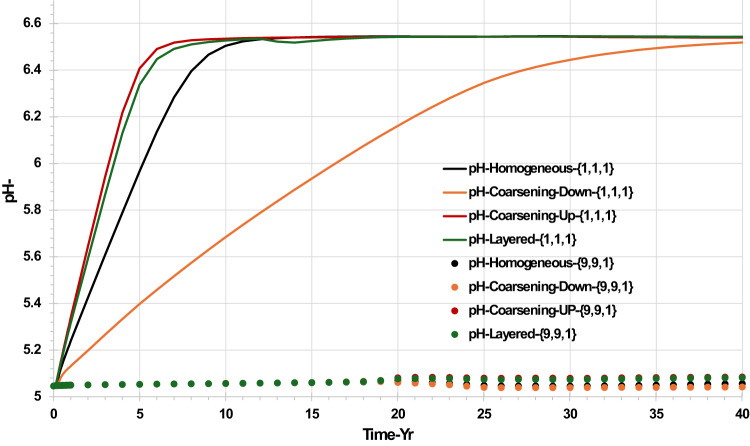
pH behaviour in the top layer injector grid block {1,1,1} and the top layer producer grid block {9,9,1} versus time—permeability distribution scenarios.

Figure 7 shows the resulting pH behaviour in the top layer around the injector, grid {1,1,1}, and in the top layer around the producer, grid {9,9,1}, for all permeability distribution scenarios. From the figure, it may be observed that the pH values exactly reflect the changes in CO_2_ concentration in the water phase in the same blocks, shown in Fig. 6. pH values in the producer block remain around 5.05 to 5.10 for all scenarios, while the pH values in the injector block increase from 5.05 to 6.75, depending on the availability of CO_2_.

**Fig. 8:**
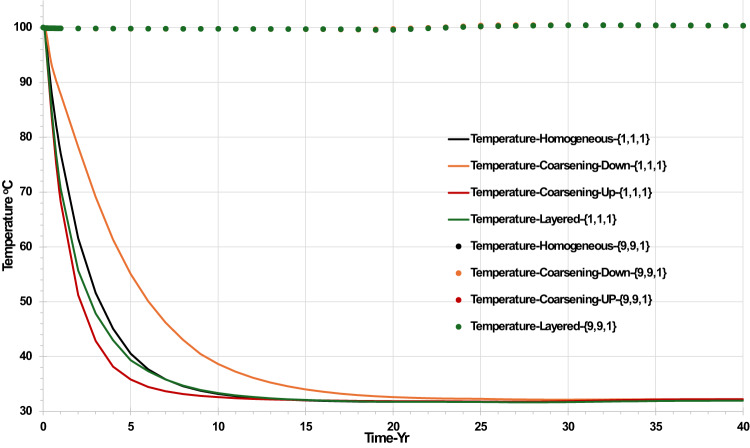
Temperature behaviour in the top layer injector grid block {1,1,1} and the top layer producer grid block {9,9,1} versus time—permeability distribution scenarios.

Figure 8 shows that the temperature in cells near the injector is impacted by the permeability distribution, whereas the cooling front does not reach the producer well during the lifetime of the calculation, so temperatures in the vicinity of the producer do not change, regardless of permeability distribution. Cooling occurs around the injection well because the temperature of the injection water is 40 ℃, while the reservoir temperature is 100 ℃. However, the rate at which the first cells cools depends on the volume throughput. The rock around the injection well will eventually be cooled from the initial reservoir temperature to the bottom hole temperature of the injection well. The cooling time is variable and depends on the impact of heterogeneity on the volume throughput at the location where the temperature is being evaluated. Figure 8 shows that the earliest temperature drop in the top layer (Layer 1) happened in the coarsening-up scenario, the cooling effect taking 10 years to be fully established. On the other hand, it takes over 25 years for the colling effect to reduce the cell temperature in the top layer to the injection water temperature in the coarsening down scenario. The difference in timing is simply due to the coarsening up scenario having a much higher volume throughput in the top layer where the temperature is being evaluated than does the coarsening down scenario.

This decrease in temperature impacts the solubility of CO_2_ in water (increasing it), and also the solubility of calcite and dolomite (increasing them both), as well as having a significant effect on the behaviour of anhydrite and gypsum, as will be noted below.

### Impact of mineral reactions

In this section of the study, the model is extended to include aqueous and mineral geochemistry, which also addresses the impact of dissolved CO_2_. A particular focus of the study is the impact of magnesium-rich carbonate mineral precipitation, since its impact on sulphate scaling is not well understood. (We refer to the magnesium-rich carbonate mineral as “dolomite” in the remainder of our description for convenience in terms of the modelling.) The concentrations of ions in the formation and injection waters used in this 3D model are shown in Table 5, and are based on brine compositions that may be encountered in the Santos Basin (Martins and Mackay, 201425); the formation water equilibrated with the minerals present and the CO_2_ content in the oil, and the injection water has the composition typical of full sulphate seawater.

**Table 5:** Ion concentrations in formation and injection waters.

Ions	Formation water (mg/L)	Injection water (mg/L)
Na^+^	10,768	10,679
Mg^2+^	73	1300
Ca^2+^	2177	405
Ba^2+^	604	0
Sr^2+^	8	8
Cl^−^	20,193	19,168
SO_4_^2−^	0	2780
HCO_3_^−^	470	154
pH	5.0	8.1

**Fig. 9:**
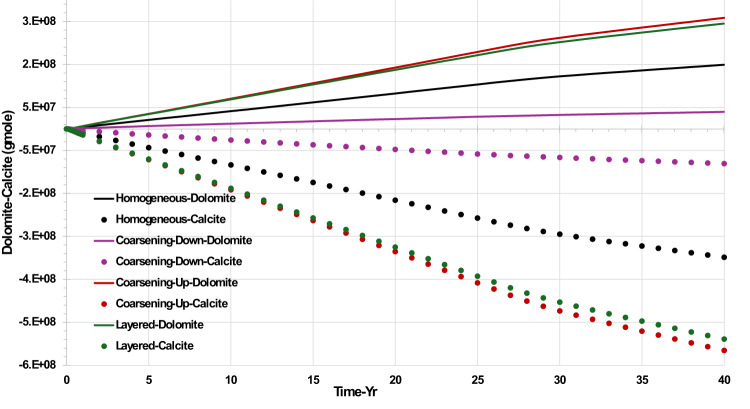
Dolomite (precipitation) and calcite (dissolution) behaviour in the top layer injector grid block {1,1,1} versus time—permeability distribution scenarios.

From Fig. 9 it is clear that in all four scenarios, calcite dissolution is coupled with dolomite precipitation, reflecting the stoichiometry and equilibrium dynamics of the system. Dolomite precipitation requires one mole of Mg^2^⁺ (supplied by seawater), one mole of Ca^2^⁺ (from calcite dissolution), and two moles of CO_3_^2^⁻. Since each mole of calcite contributes one mole of Ca^2^⁺ and one mole of CO_3_^2^⁻, the formation of one mole of dolomite necessitates the dissolution of two moles of calcite. This stoichiometric relationship explains the observed increase in aqueous Ca^2^⁺ concentration during dolomite formation.

While this calcite–dolomite coupling dominates the carbonate chemistry in injection block {1,1,1}, the system includes additional mineral reactions involving Ca^2^⁺, particularly under variable CO_2_ concentrations and temperature conditions. Therefore, although the calcite–dolomite interaction is central, it does not occur in isolation from other geochemical processes affecting calcium availability.

Figure 9 shows the lowest dolomite precipitation and lowest calcite dissolution in the injector block {1,1,1} in the coarsening down scenario, since this is the scenario with the lowest volume throughput in the top layer of the model. In fact, the various profiles of dolomite precipitation, and the various profiles of calcite dissolution, would collapse onto each other if plotted against local cell PV throughput, rather than against time. This is important, since it identifies that the extent of the reactivity is not determined by the extent to which the injected brine mixes with the formation brine—as is often the case with barite precipitation, say—but it depends on the PV throughput of injected brine coming into contact with the calcite in the rock matrix. That is to say, the reactions are controlled not by brine-brine mixing interactions, but by brine-rock contact interactions.

**Fig. 10:**
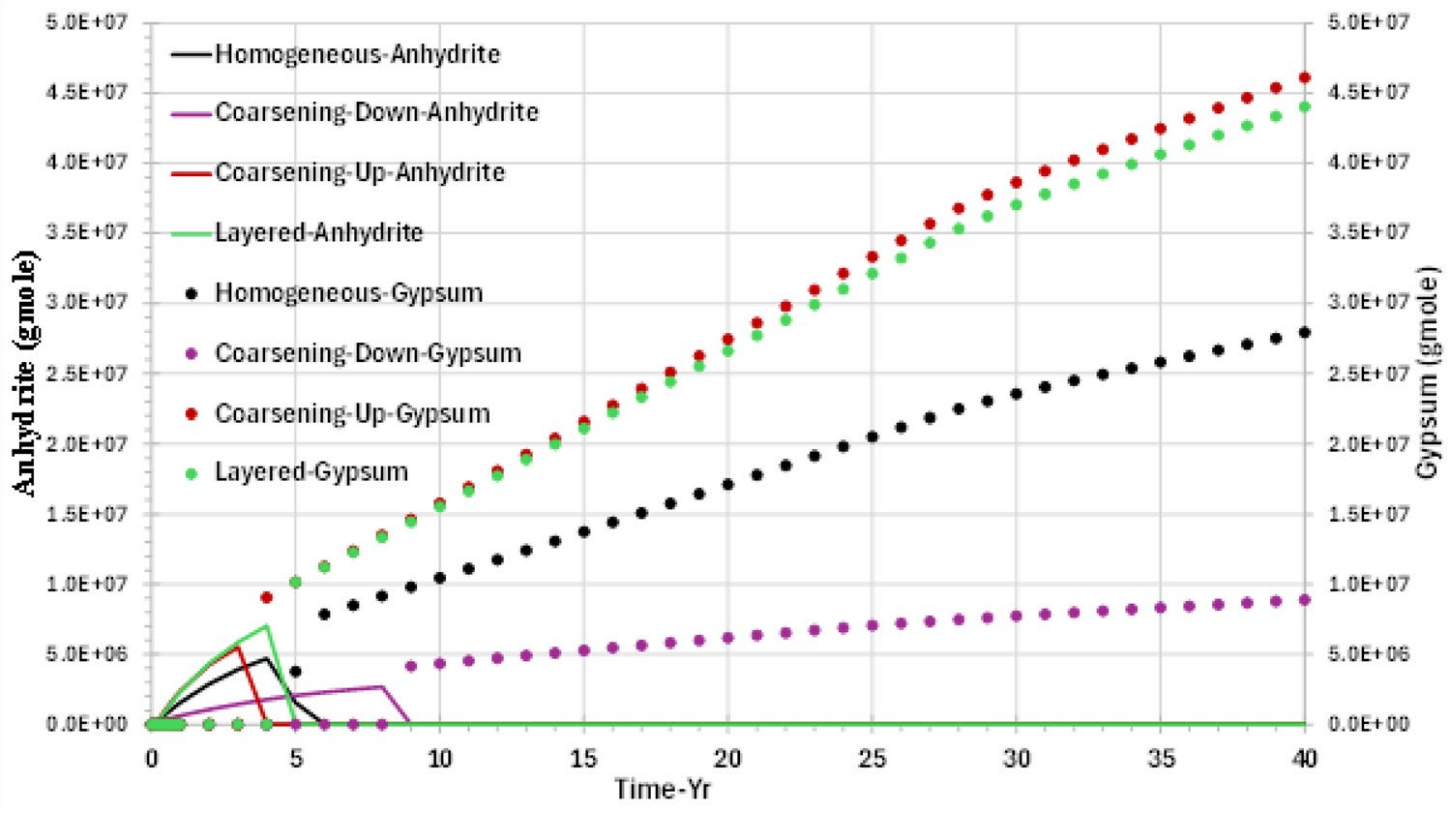
Anhydrite and gypsum precipitation behaviour in the top layer injector grid block {1,1,1} versus time—permeability distribution scenarios.

Figure 10 shows the behaviour of both anhydrite and gypsum in block {1,1,1}. Anhydrite precipitation begins immediately at the beginning of the waterflood, with one mole of anhydrite consuming one mole of sulphate from seawater and the excess mole of calcium when two moles of calcite dissolve.

Anhydrite precipitation decreases to zero when the temperature in the cell drops below 40 °C, as mentioned above, depending on the cooling time for each scenario (see Fig. 8). At that temperature, gypsum solubility becomes lower than anhydrite solubility, meaning that as the system cools, the precipitation of gypsum replaces the precipitation of anhydrite—but maintaining a very similar trend. Thus, the excess calcium from the calcite dissolution goes from being involved in anhydrite precipitation to being involved in gypsum precipitation—at about the same rate of deposition. The figure shows the maximum gypsum precipitation taking place in both scenarios, layered and coarsening-up models.

**Fig. 11:**
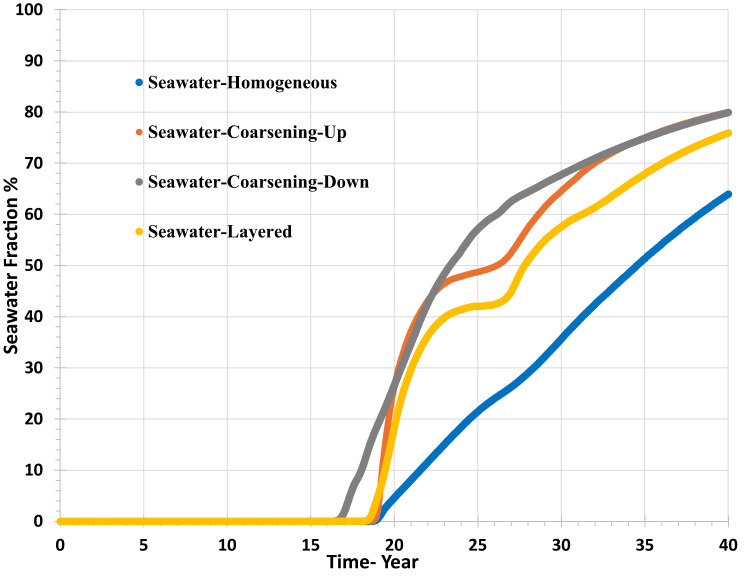
Sea water fraction (%) in the producer well versus time—permeability distribution scenarios.

Figure 11 shows us that the breakthrough and the increase in seawater fraction in the producer well depends on the permeability distribution, the earliest seawater breakthrough being around year 17 (coarsening-down scenario) and the latest being around year 19–20 for the rest of the scenarios. This means there is a significant impact of mixing and of the chemical reactions during the brine displacement through the porous medium. The maximum value of seawater fraction at the producer is only 65–80% at the end of the simulation. Furthermore, water cut increases over time, so for the same produced water composition, the deposition rate would be higher later than earlier, and the deposition rate depends on both saturation ratio, SR, and water flow rate, as seen below.

**Fig. 12:**
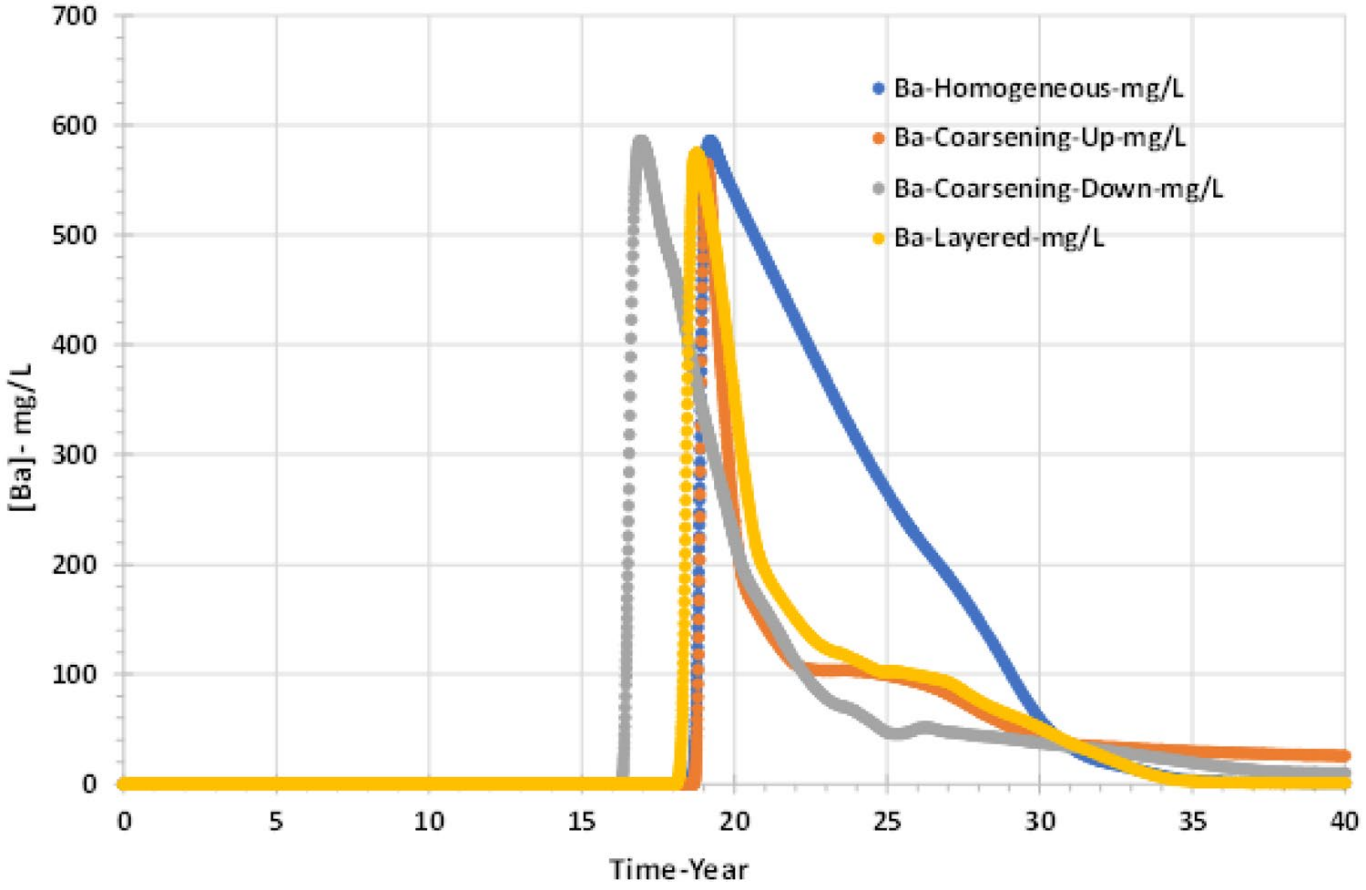
Barium concentrations in the producer well versus time—permeability distribution scenarios.

In Fig. 12 it can be seen that at the same time as the seawater breakthrough, there is a drop in the concentration of barium. The anhydrite reaction consumes the initially injected sulphate ions, to the extent that there are no sulphate ions left in the breakthrough brine to react with the barium. Thus, the decrease in barium is purely due to the dilution of the formation water by seawater.

This means that the chemical reactions indirectly reduce the barite scaling risk in the production wells as the anhydrite reaction deep within the reservoir prevents the breakthrough of sulphate until much later. Also, the figure shows the coarsening down model has the earliest effect on the barium ion behaviour, and the homogenous scenario has kept the concentration of barium high as long as possible.

**Fig. 13:**
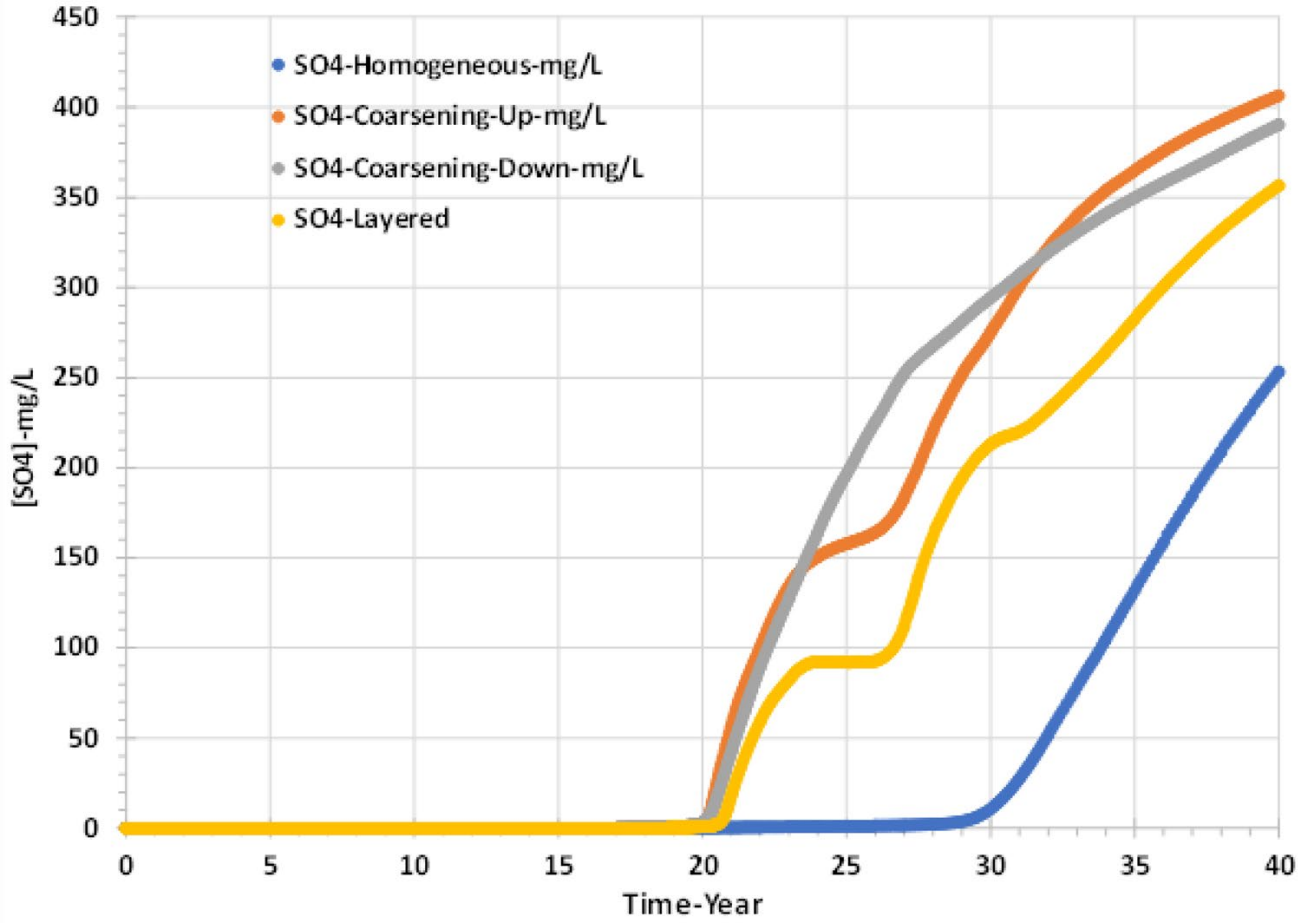
Sulphate concentrations in the producer well versus time—permeability distribution scenarios.

Figure 13 illustrates the behaviour of sulphate concentration in the producer well for all four scenarios. As expected, the sulphate concentration increases as the injection water fraction increases, but not as quickly as might otherwise be expected due to the precipitation of anhydrite (and then gypsum). Also, the sulphate increases over time differently for each scenario: early sulphate breakthrough occurs in the three heterogeneous scenarios and the latest sulphate breakthrough occurs in the homogenous model.

**Fig. 14:**
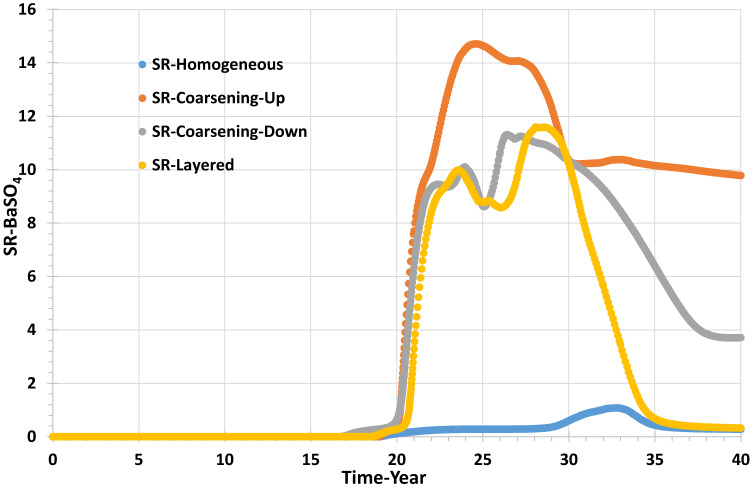
Barite Saturation Ratio in the producer well versus time—permeability distribution scenarios.

Figure 14 shows that the Saturation Ratio (SR) in the producer well becomes non-zero at seawater breakthrough, and the peak value depends on the extent of *in* situ mixing in each model because, as mentioned previously for Figs. 11, 12 and 13, the produced barium and sulphate concentrations are dependent not only on the seawater fraction profile, but also on the amount of mixing of injected and formation brines occurring in the inter well regions, and on the depletion in scaling ions that occurs due to precipitation deep within the reservoir.

**Fig. 15:**
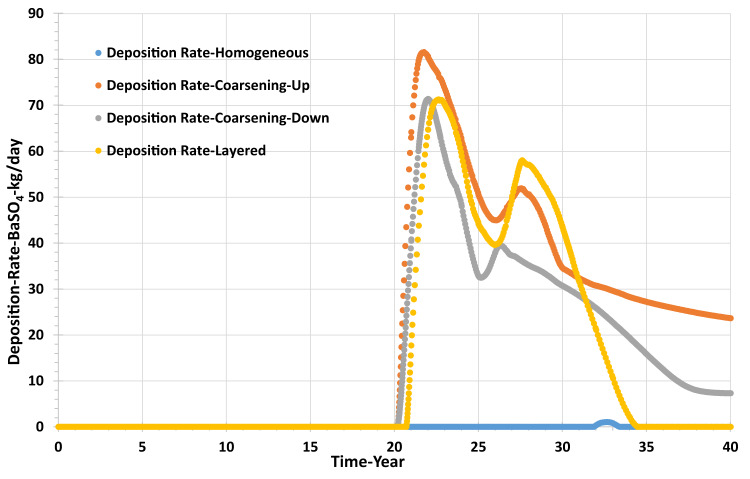
Barite deposition rate in the producer well versus time—permeability distribution scenarios.

Figure 15 shows the barite precipitation rate in the producer well; deposition occurs whenever SR > 1, which is after year 20, but the deposition rate is strongly influenced by the abundance of the two scaling ions—even for a given SR—and so is related to the amount of mixing that has taken place, which is strongly influenced by the degree and type of heterogeneity. Figure 15 shows the maximum barite deposition rate occurred in the coarsening-up model, about 80 kg/day, and the lowest precipitation rate was calculated in the homogenous model, with just around 2 kg/day.

### Effect of grid resolution

The effect of a reservoir model’s spatial discretization on simulation outcomes is known as the "Grid Resolution Effect." The effect occurs because of the level of detail or resolution in the numerical grid. In this section of the study, the impact of grid resolution on the results is contrasted with the differences observed above due to the permeability distribution effect. The four scenarios included in this section, as mentioned in Table 4, are all homogenous, with the number of grid cells in the I, J, and K directions being (9, 9, 4), (90, 90, 40), (45, 45, 20), and (27, 27, 12). Grid cell sizes are reduced correspondingly to ensure the overall model size in each direction remains constant.

**Fig. 16:**
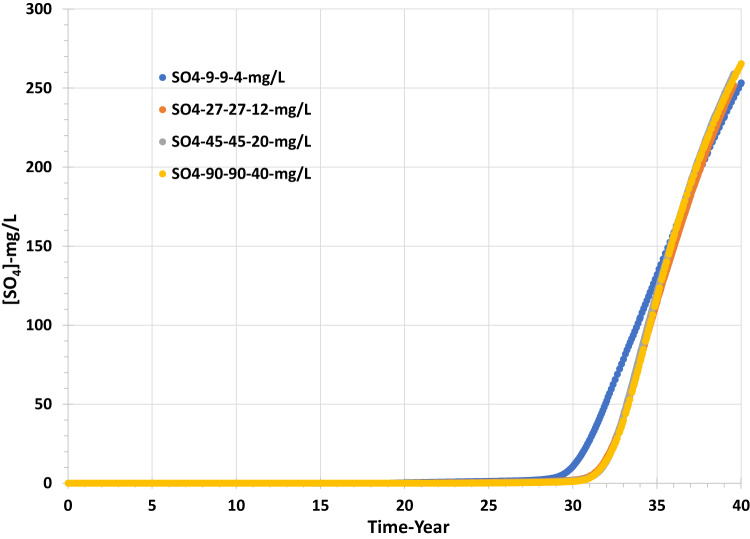
Sulphate concentration in the producer well versus time—grid resolution scenarios.

**Fig. 17:**
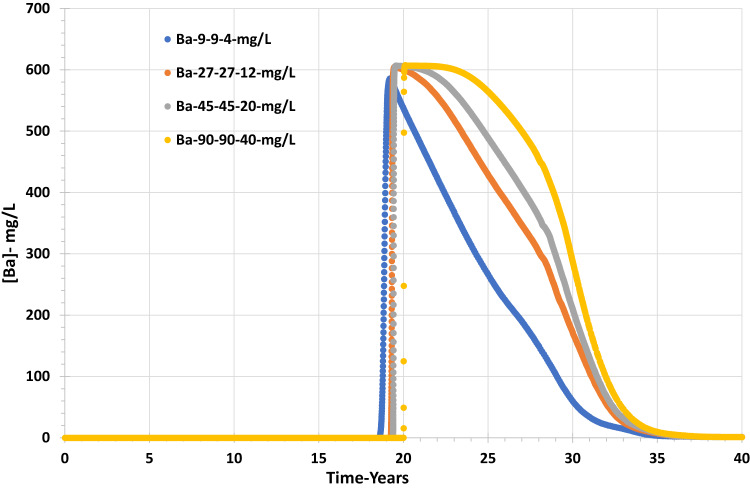
Barium concentration in the producer well versus time—grid resolution scenarios.

**Fig. 18:**
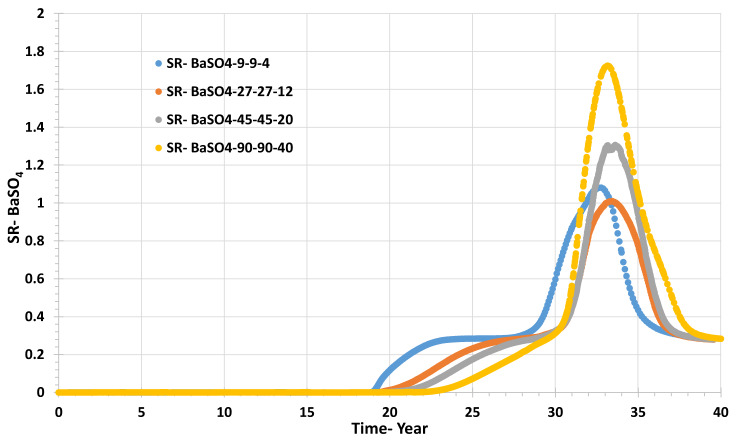
Barite Saturation Ratio in the producer well versus time—grid resolution scenarios.

Figures 16 and 17 show that the grid resolution sensitivity analysis has the lowest effect on the behaviour of barium and sulphate concentrations and, thereafter, the values of saturation ratio and deposition rate. Figure 16 shows that the sulphate concentration increases similarly for all four scenarios, although the sulphate breakthrough time appears to be earlier by about two years in the coarsest model compared to the other three, which all suggest sulphate breakthrough after around year 30. By contrast, in the sensitivity calculation to permeability distribution, sulphate breakthrough time was much more variable, with a wider range for apparent sulphate breakthrough, by about ten years. Although sulphate breakthrough is often associated with seawater breakthrough, it is not necessarily the best indicator of seawater breakthrough, since sulphate will be consumed by barite precipitation reactions deep in the reservoir. Greater numerical dispersion in coarser cells will tend to exaggerate the degree of brine mixing and hence exaggerate the degree of barite precipitation in situ, and hence it will exaggerate the delay in sulphate breakthrough. A better measure of seawater breakthrough is, in fact, the measured barium concentration, since as soon as seawater breaks through, the barium concentration will decrease due to dilution of the formation water by seawater. Any barite precipitation will act to further reduce the barium concentration—but only once some seawater has broken through. A cursory glance at Fig. 16 would suggest seawater breaks through around year 30, with a couple of years of variation depending on the grid resolution. However, Fig. 17 suggests barium starts to decline between years 18 and 20 (depending on resolution), a decade earlier, and thus, we identify seawater breakthrough is, in fact, a decade earlier than the sulphate plots suggest. Careful examination of Fig. 18 identifies that the Saturation Ratio for barite starts to increase around year 20, not year 30, indicating the earlier date for seawater breakthrough, despite the scaling risk not becoming apparent (SR > 1) till year 32 at the earliest.

Figure 18 shows the behaviour of SR for different grid resolution scenarios, with the maximum SR being around 1.8 in the highest resolution model, decreasing to just above unity in the lowest resolution model.

**Fig. 19:**
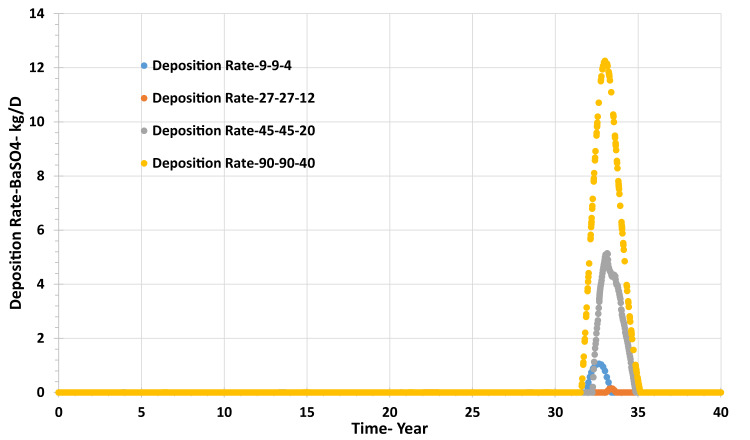
Barite deposition rate in the producer well versus time—grid resolution scenarios.

The deposition rate in the grid resolution tests is also very low, being a maximum of 12 kg/day in the highest resolution model, and dropping to almost nil in the lowest resolution model (Fig. 19).

## Discussion and conclusions

In this study, using a synthetic two-well 3D 3-phase model, we have investigated the impact of variations in pressure and temperature on fluid phase behaviour, including the evolution of gas as pressure initially drops below bubble point pressure and the impact these behaviours have on geochemical reactions. By their nature, such modelling activities are designed to provide insights as to the type of behaviours that should be anticipated in a range of reservoir types—in this case, carbonate reservoirs containing crude oil with a finite CO_2_ content subjected to seawater flooding. The modelling is not intended as a precise prediction of a specific system. The coarseness of the model means that the reservoir description is not detailed, but, more importantly, that numerical dispersion effects will be observed. However, as the key processes are due to brine interactions with immobile phases (the rock and residual oil), the numerical errors that arise during interactions between mobile fluid phases do not critically impact the conclusions. This can be seen from the sensitivity studies to grid resolution and permeability distribution, where there is an impact due to the choice of grid resolution clearly evident, but the impact due to permeability distribution on the timing of scaling risk events is much more evident.

In the homogeneous model, there is a significant degree of brine mixing in the reservoir, which results in a significant consumption of sulphate ions, preventing the sulphate concentration in the producer from rising to sufficient levels to cause scaling in the producer until some ten years after the barium concentration has started to decline. By this point there is still some barium in the produced brine, but it is much lower than initially, and so the scaling risk when the sulphate is produced is much lower. However, in the heterogenous layered cases, seawater breaks through in the high permeability layers (assisted by gravity in the coarsening down case), somewhat earlier, and importantly, the amount of mixing with formation water in the reservoir is reduced, limiting the depletion of sulphate ions. Thus, there is coproduction of sulphate ions form the high permeability layer and barium ions from the low permeability layers yet to experience seawater breakthrough, and so in the heterogenous cases the mixing takes place *inside* the well rather than in the reservoir, creating the much higher scaling risk for the well, and for longer.

Geochemical reactions are limited to the principal ones observed in such subsurface reservoirs, and while the equations and input parameters may continually be improved, choices were made to ensure the best available models were selected for the specific conditions being modelled. The principal point is that the key behaviours and qualitative outcomes should be taken into consideration when addressing such systems—albeit more precise models will be required for any specific reservoir for which decision-making around injection water quality, flow rates, and reservoir management options need to be determined.

We identify that around the production well, the presence of free gas impacts the availability of CO_2_ to partition into the brine, and this will impact the extent to which calcite dissolves or precipitates. Eventually, as the evolved gas re-dissolves back in the oil phase as pressure rises again, the availability of CO_2_ for partitioning into the brine phase declines, the pH increases, and any calcite dissolution in the reservoir or precipitation around and in the producer will decline. (It should be noted that increase of dissolution in the formation may be beneficial in terms of productivity, but that this also means there is a higher concentration of scaling ions remaining in the brine as it is produced, and when the CO_2_ evolves inside the well and production system, there may be a higher risk of scale precipitation there. Evidently, this scale will be easier to manage than scale formation within the reservoir since, depending on where in the well the CO_2_ evolves, it may be possible to control by means of continuous injection of scale inhibitor.

Around the injection well, there is a contrasting story. As the injection water contains almost no CO_2_, as it flows past residual oil, it will strip CO_2_ out of the residual oil until, eventually, around the injection well, the CO_2_ in the residual oil will become completely depleted. This CO_2_ will again keep scaling ions in the solution and, indeed, may drive the dissolution of calcite. As the injection brine in this system is undersaturated with respect to calcite, regardless of the partitioning of CO_2_, calcite dissolution will take place over the entire injection period. However, when the CO_2_ becomes depleted, the relatively higher concentration of magnesium in the injection brine means the brine will become oversaturated with respect to dolomite, which will start to deposit. Thus, there will eventually be a coupled process of calcite dissolution and dolomite precipitation around and away from the injection well. This process may cause some change in porosity, but there is no evidence in this work that that change will be significant. What is significant is that for each mole of dolomite that precipitates, there must be two moles of calcite dissolved. The mole of dolomite precipitation will consume one mole of magnesium (injected in the seawater—so this reaction pair only occurs where seawater has broken through), two moles of carbonate (from the two moles of calcite dissolved), and one mole of calcium (from one of the moles of calcite dissolved). This leaves one mole of calcium in solution, a consequence of primary mineral dissolution, and is now available to react with sulphate injected in the seawater. Thus, for every mole of magnesium stripped out of the seawater by the dolomite reactions, there will be one mole of sulphate stripped out due to the anhydrite reaction. The impact of cooling in the near well zone is that the anhydrite reaction, which occurs at higher temperatures, will be replaced by gypsum precipitation at injection well bottom hole temperatures.

This intricate series of reactions has an overall impact of stripping some (although not all) of the sulphate out of the seawater. This then has an impact on barite precipitation reaction around the production well, which still occurs, but is significantly delayed by these in situ reactions. This also raises the question of whether or not desulphation of injection water is beneficial under these circumstances—why undergo the effort and expense of treating the injection water if the reservoir itself is going to naturally deplete the sulphate ions, limiting the scaling risk at the producer wells?

## Data Availability

The datasets used and/or analysed during the current study available from the corresponding author on reasonable request.

## Abbreviations

3D: Three dimensional
Ba^2+^: Barium
BaSO_4_: Barium sulphate
Ca^2+^: Calcium
CaSO_4_: Calcium sulphate
CMG: Computer Modelling Group software
FSSW: Full sulphate seawater
GEM: Leading reservoir simulation software for compositional, chemical, and unconventional Equation of State (EoS) based reservoir modelling
H P V Throughput: Hydrocarbon pore volume throughput
LSSW: Low Sulphate Seawater
PV: Pore volume
SO_4_^2^^−^: Sulphate
Sr^2+^: Strontium
SrSO_4_: Strontium sulphate
SR: Saturation ratio
